# Recombinant production of a diffusible signal factor inhibits *Salmonella* invasion and animal carriage

**DOI:** 10.1080/19490976.2023.2208498

**Published:** 2023-05-09

**Authors:** Mudasir Ali Rather, Rimi Chowdhury, Paulina D. Pavinski Bitar, Craig Altier

**Affiliations:** Department of Population Medicine and Diagnostic Sciences, Cornell University, Ithaca, NY, USA

**Keywords:** *Salmonella*, invasion, diffusible signal factor, *cis*-2-hexadecenoic acid, *rpfF*, virulence, *E. coli* Nissle 1917, chickens

## Abstract

The complex chemical environment of the intestine is defined largely by the metabolic products of the resident microbiota. Enteric pathogens, elegantly evolved to thrive in the gut, use these chemical products as signals to recognize specific niches and to promote their survival and virulence. Our previous work has shown that a specific class of quorum-sensing molecules found within the gut, termed diffusible signal factors (DSF), signals the repression of *Salmonella* tissue invasion, thus defining a means by which this pathogen recognizes its location and modulates virulence to optimize its survival. Here, we determined whether the recombinant production of a DSF could reduce *Salmonella* virulence *in* *vitro* and *in* *vivo*. We found that the most potent repressor of *Salmonella* invasion, *cis*-2-hexadecenoic acid (c2-HDA), could be recombinantly produced in *E.* *coli* by the addition of a single exogenous gene encoding a fatty acid enoyl-CoA dehydratase/thioesterase and that co-culture of the recombinant strain with *Salmonella* potently inhibited tissue invasion by repressing *Salmonella* genes required for this essential virulence function. Using the well characterized *E.* *coli* Nissle 1917 strain and a chicken infection model, we found that the recombinant DSF-producing strain could be stably maintained in the large intestine. Further, challenge studies demonstrated that this recombinant organism could significantly reduce *Salmonella* colonization of the cecum, the site of carriage in this animal species. These findings thus describe a plausible means by which *Salmonella* virulence may be affected in animals by *in* *situ* chemical manipulation of functions essential for colonization and virulence.

## Plain Language Summary

Despite our best efforts, infections of agricultural animals with *Salmonella* persist, posing threats to food safety. Few, if any, measures have proven effective in reducing *Salmonella* carriage in animals used for food, a major source of this pathogen. Antibiotics are ineffective at curtailing infection and have served only to exacerbate the global crisis of antimicrobial resistance. The alternative then is to seek novel means to reduce *Salmonella* disease and carriage by preventing its colonization of livestock and poultry. Here we describe an approach targeting invasion, a function essential for *Salmonella* carriage and disease in animals. We show that a potent chemical inhibitor of invasion, the diffusible signal factor *cis*-2 hexadecenoic acid, can be produced by recombinant *E.* *coli* strains capable of stably colonizing the animal intestine, providing a means to directly affect the virulence of *Salmonella* within an animal host. These studies may thus provide a route to reduce the carriage of this pathogen in production animals and thus the spread of disease to humans.

## Introduction

*Salmonella* is exquisitely adapted to survive and proliferate within the intestines of animals. Its host range is large and diverse, including numerous poultry and livestock species in which it rarely produces clinical signs of disease, and thus presents a surreptitious threat to human health. Public health measures intended to reduce salmonellosis in people have had only limited success, largely due to the difficulty in reducing *Salmonella* contamination in animal-sourced foods.

One proposed approach to control *Salmonella* infections is based not upon killing the pathogen, as would an antimicrobial, but instead by inhibiting some function essential to the completion of its infectious lifecycle. An attractive target of intervention is invasion, the process by which *Salmonella* penetrates the epithelial cells lining the intestinal tract,^1^ a function needed for both effective colonization of the gut and disease.^2–5^ Invasion requires the coordinated expression of multiple virulence genes, controlled through a complex cascade of genetic and environmental factors.^6–10^

Among the chemical constituents of the gut known to control invasion are fatty acids, produced as metabolic products by the intestinal microbiota.^11–13^ Fatty acids typically serve as sources of nutrition for both members of the microbiota and the animal host, but one class of molecules that profoundly reduces *Salmonella* invasion is instead employed as a quorum-sensing signal by the bacterial species that produce it. Termed diffusible signal factors (DSFs), these molecules constitute a family of related long-chain fatty acids that harbor a characteristic *cis*-2 unsaturation and are used by several bacterial species to recognize quora.^14–19^ Intriguingly, *Salmonella* also senses these signals, but does so in an unconventional way. The most potent of these, *cis*-2 hexadecenoic acid (c2-HDA), binds to three *Salmonella* transcriptional activators, HilD, HilC, and RtsA, preventing their induction of genes essential for invasion.^20–22^ As c2-HDA has been isolated from the murine intestine,^21^ it presents a plausible means by which *Salmonella* recognizes the gut environment to modulate its virulence and maximize its reproductive potential.

In moderation, c2-HDA and other chemical repressors of invasion likely benefit *Salmonella* by balancing the costly expression of virulence determinants with those needed for replication and survival. Would it be possible, however, to use such chemical signals as a practical means to control *Salmonella* colonization of animals? Such an approach would require that artificially high concentrations of these signals be maintained in the gut, effectively eliminating invasion and thus preventing efficient colonization. To address this question, here we tested whether c2-HDA can be produced by a recombinant organism that stably colonizes the gut. We found that this chemical signal can be produced and excreted by *E. coli* in sufficient concentration to potently inhibit invasion in neighboring *Salmonella*, and that oral administration of this recombinant organism to chickens was able to reduce the extent of *Salmonella* colonization.

## Results

### Recombinant expression of *rpfF* reduces the expression of *Salmonella* invasion genes

**Figure 1.**
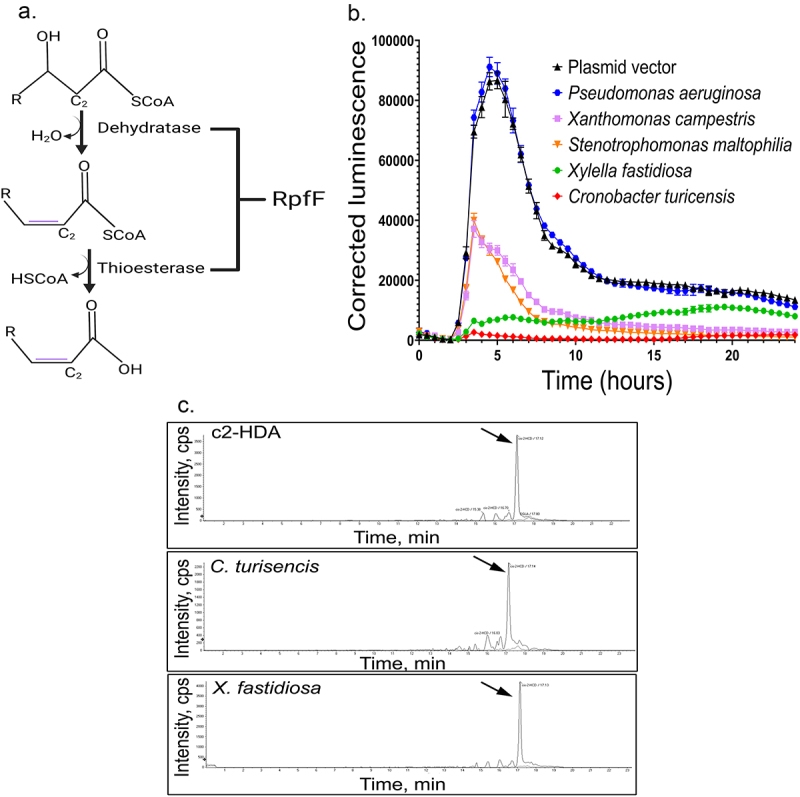
Recombinant expression of rpfF produces a diffusible signal factor that reduces the expression of *Salmonella* invasion genes. A. RpfF encodes dehydratase and thioesterase functions to produce *cis*-2 unsaturated fatty acids (c2-HDA), from products of long-chain fatty acid biosynthesis. B. Orthologs of *rpfF* from the species shown were expressed in *E.* *coli* DH5α on a multi-copy plasmid in co-culture with *S.* Typhimurium carrying a plasmid-borne *hilA-luxCDABE* reporter fusion, and *hilA* expression was measured by luminescence as a function of culture turbidity. Error bars show standard deviation (*n* = 5). C. LC-MS was used to analyze fatty-acid extracts of culture supernatants from *E.* *coli* strains expressing *rpfF* from *C.* *turicensis* and *X.* *fastidiosa*, with both demonstrating a peak at 17 minutes, consistent with c2-HDA.

The production of DSFs utilizes the ubiquitous bacterial pathway for the synthesis of long-chain fatty acids, with the addition of a single dehydratase/thioesterase enzyme that introduces the *cis*-oriented double bond at the second carbon that is characteristic to these quorum-sensing signals (Figure 1a).^16^ To determine whether c2-HDA or related signaling compounds could be recombinantly produced, we introduced into *E. coli* orthologous genes encoding this dehydratase/thioesterase (most typically named *rpfF*) from several bacterial species. The genes were codon-optimized for improved translation in *E. coli* and cloned onto a multi-copy plasmid vector under the control of the hybrid *tacI* promoter. We then tested the ability of the recombinant strains to reduce the expression of *Salmonella* invasion genes by growing them in co-culture with a *Salmonella* serovar Typhimurium strain harboring a *hilA-luxCDABE* reporter fusion,^12^ measuring the expression of this transcriptional activator essential to invasion. Using the recombinant *E. coli* provided at a ratio of 1:10 to *Salmonella*, we found a range of effects, depending upon the origin of the gene (Figure 1b). The *rpfF* orthologs of *Cronobacter turicensis* and *Xylella fastidiosa* were both highly effective in repressing *hilA*, reducing its peak expression by 33- and 11-fold, respectively. The *rpfF* homolog orthologs of *Xanthomonas campestris* and *Stenotrophomonas maltophilia* were similar to each other in their efficacy and notably less effective, reducing peak *hilA* expression by ~twofold. The *Pseudomonas aeruginosa rpfF* ortholog, in contrast, had no discernable effect, with *Salmonella hilA* expression being unchanged from that found in co-culture with *E. coli* harboring the plasmid vector without *rpfF*. To determine whether these recombinant strains produced a diffusible factor, and to identify it, we used liquid chromatography–mass spectrometry (LC-MS). Analysis of culture supernatant from the two most potent strains, expressing *rpfF* from *C. turicensis* and *X. fastidiosa* under the control of promoters known to function efficiently in *E. coli*,^23^ showed both to be producing c2-HDA, based upon spectral comparison to commercially available purified samples of this compound (Figure 1c). These results thus demonstrate that the expression in *E. coli* of a single gene encoding an enzyme required for DSF production induced the recombinant strain to produce and excrete sufficient c2-HDA to potently repress an essential *Salmonella* invasion determinant, even when the proportion of that recombinant strain within the population was low.

### *E.* *coli* can stably produce c2-HDA

**Figure 2.**
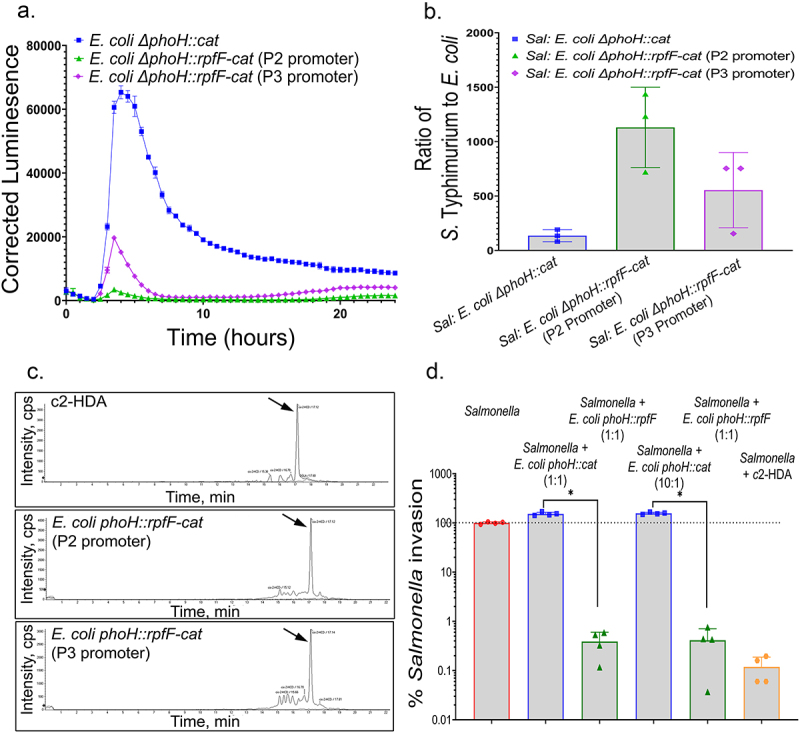
Stable production of c2-HDA by *E.* *coli* reduces expression of *Salmonella* invasion genes and cell invasion. A. *rpfF* of *C.* *turicensis* under the control of promoters of different strengths (P3 promoter for high expression; P2 promoter for medium expression) was integrated into the genome of *E.* *coli* Nissle 1917, and strains were grown in co-culture with *S.* Typhimurium carrying a plasmid-borne *hilA-luxCDABE* reporter fusion. *hilA* expression was measured by luminescence as a function of culture turbidity. Error bars show standard deviation (*n* = 5). B. The ratio of *Salmonella* to *E.* *coli* after 24 hours of co-culture was determined by selective plating on MacConkey lactose agar (*n* = 3). C. LC-MS was used to analyze fatty-acid extracts of culture supernatants from *E.* *coli* strains expressing *rpfF*, with both high- and medium-expressing constructs demonstrating a peak at 17 minutes, consistent with c2-HDA. The c2-HDA panel is identical to that shown in Fig. 1B, reproduced here for reference. D. *Salmonella* invasion was determined by measuring its penetration of cultured HEp-2 epithelial cells when grown in co-culture with *E.* *coli* expressing *rpfF* under the control of the P3 promoter or an isogenic strain lacking *rpfF*. The ratio of *Salmonella* to *E.* *coli* in the inoculum is shown for each strain combination. *Salmonella* grown alone with the addition of either 20 µM c2-HDA or an equal volume of DMSO were used as controls. Results are shown as the proportion of invasion as compared to *Salmonella* alone grown with DMSO; mean invasion of this strain was 2.4% of the inoculum. Error bars show standard deviation (*n* = 4).

We next sought to determine whether c2-HDA could be reliably produced by a recombinant bacterium capable of sustained carriage in the intestines of animals. For this purpose, we chose *E. coli* Nissle 1917, a strain that has been safely administered to both humans and animals.^24–28^ *rpfF* of *C. turicensis* was integrated into the *E. coli* chromosome in *phoH*, a pseudogene of this strain. Using constructs with promoters of varying strengths,^23^ we tested both their ability to produce c2-HDA and their effects on the growth of the recombinant strain, again using an initial ratio of 1:10 *E. coli* to *Salmonella*. Constructs driven by promoters reported to be of both high- and medium-strength demonstrated substantial effects on the expression of *Salmonella* invasion genes, reducing peak expression of *hilA* by 6- and 22-fold, respectively, when grown in co-culture with the recombinant strain, as compared to growth with the isogenic nonrecombinant control strain (Figure 2a). To ensure that the observed repressive effects were not simply the result of the *E. coli* strains out-competing *Salmonella* in co-culture, we measured the proportion of the two species after 24 hours of co-incubation by selective plating (Figure 2b). For the nonrecombinant control strain, the ratio of *E. coli* to *Salmonella* was substantially reduced during the assay to 1:130. The proportions of the two *rpfF*-expressing strains were even more greatly reduced to 1:>500 for the high-strength promoter construct and 1:>1100 for the medium-strength construct, demonstrating their efficacy when present in low abundance. Again using LC-MS, we confirmed that the product of these strains was indeed c2-HDA (Figure 2c). To determine whether this gene repression resulted in a loss of invasion, we tested the ability of recombinant *E. coli* expressing *rpfF* to inhibit the invasion of cultured HEp-2 epithelial cells by *Salmonella* when the two were grown in co-culture. Strains were initially grown in pairs under conditions that produced an excess of *Salmonella* to *E. coli* of~3:1. We found that the *rpfF*-expressing strain profoundly impaired *Salmonella* invasion, reducing it by 390-fold, compared to that of *Salmonella* grown without *E. coli* (Figure 2d). The non-recombinant strain, however, induced no invasion defect. As a more stringent test, we grew pairs of strains in ratios that produced an even greater difference between the two: 27:1 *Salmonella* to *E. coli* for the *rpfF*-expressing *E. coli* and 26:1 for its non-recombinant isogenic derivate. Again, *Salmonella* invasion was severely reduced by growth with the recombinant strain, 380-fold, while the non-recombinant control strain had no effect. These results, taken together, thus demonstrate that single-copy chromosomal expression of *rpfF* in *E. coli* has profound effects on *Salmonella* cell invasion through its repression of genes required for this essential virulence function, even when the recombinant organism represents only a small fraction of the population.

**Figure 3.**
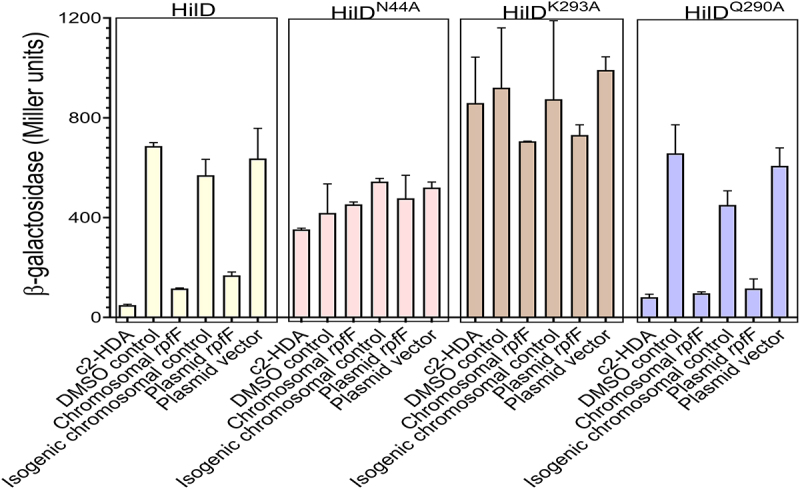
Recombinantly produced c2-HDA inhibits *Salmonella* invasion through its effects on the transcriptional activator HilD. *E.* *coli* strains expressing *rpfF* of *C.* *turicensis* were co-cultured with *S.* Typhimurium carrying a *sipB:lacZY* reporter fusion and one of the *hilD* alleles shown, and *sipB* expression was measured using β-galactosidase assays. As a control for plasmid-borne expression, the empty cloning vector was used, and for chromosomal expression, the isogenic strain without rpfF was used. Error bars show standard deviation (*n* = 4).

Fatty-acid signaling molecules that repress *Salmonella* invasion, including c2-HDA, function by binding to the master transcriptional activator HilD, utilizing specific amino acids of this protein that differ depending upon the chemical structure of the signal.^21^ To determine whether the recombinantly produced signal functioned in this way, we extracted fatty acids from the culture supernatant of recombinant *E. coli* expressing the *C. turicensis rpfF* ortholog. We then added these extracts to *Salmonella hilD* mutants that are unresponsive to specific classes of signals,^21^ measuring the effects on a *lacZY* reporter fusion to the invasion gene *sipB* (Figure 3). Expression of this gene was greatly reduced in a *Salmonella* strain with wild-type *hilD* by the addition of fatty acids extracted from *E. coli* recombinantly expressing *rpfF* from either a multi-copy plasmid or a single-copy chromosomal integration. A HilD^N44A^ point mutant, however, unresponsive to repression by all fatty acids, produced no loss of expression, as did a HilD^K293A^ mutant, known to be unresponsive to repression only by fatty acids with a *cis*-2 unsaturation. In contrast, a HilD^Q290A^ mutant, refractory only to fatty acids without a *cis*-2 unsaturation, demonstrated repression equal to that of wild-type HilD, thus demonstrating that the functional diffusible factor is indeed a fatty acid with a *cis*-2 unsaturation, clearly implicating c2-HDA as the cause of invasion gene repression.

### c2-HDA reduces the proportion of the *Salmonella* population expressing invasion genes

**Figure 4.**
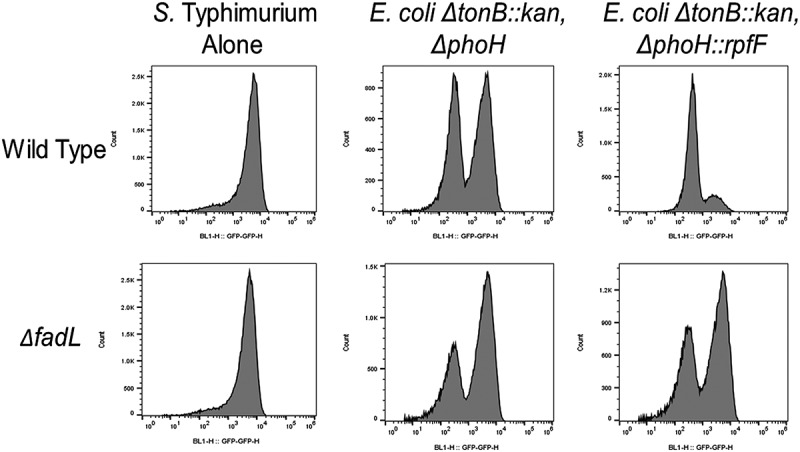
Recombinant production of c2-HDA reduces the proportion of the *Salmonella* population expressing invasion genes. A dual-fluorescent *S.* Typhimurium reporter strain (Δ*phoN*:BFP, *sicA*-GFP) was used to determine total numbers of *Salmonella* and the proportion expressing the invasion gene *sicA* when grown alone or in co-culture with isogenic *E.* *coli* Nissle 1917 Δ*tonB* strains expressing or lacking *rpfF*. *Salmonella fadL* null mutants were used to assess the importance of fatty-acid transport to the effects of c2-HDA. Numbers of BFP- and GFP-expressing *Salmonella* were determined by flow cytometry; percentage of the *Salmonella* population expressing *sicA* for each strain and condition is indicated.

In both laboratory medium and within animals, two populations of *Salmonella* exist: one expressing invasion genes at high levels and another demonstrating little or no detectable expression.^2,20,29^ The proportion of invasion-competent bacteria within this bistable population is thought to be essential to the virulence of the pathogen.^29,30^ To test whether the recombinant production of c2-HDA altered that proportion, we employed a dual-fluorescent *Salmonella* reporter strain constitutively producing BFP, used to determine the total number of *Salmonella*, and encoding the gene for GFP linked to the promoter of the invasion gene *sicA* within the chromosome,^2^ assessing *sicA* expression by flow cytometry (Figure 4). In untreated cultures grown in laboratory medium, we found the proportion of the population expressing *sicA* to be 79% after 6 hours of growth. However, the growth of the *Salmonella* reporter strain in co-culture with recombinant *E. coli* expressing *rpfF* resulted in a decrease in *sicA* expression by greater than fivefold, to 16% of the population. Co-culture with an isogenic *E. coli* strain lacking *rpfF* produced a lesser reduction of 1.5-fold, with *Salmonella sicA* expression detected in 51% of the population, but significantly less so than for the recombinant strain. If this effect were due to the acquisition of c2-HDA, a strain unable to efficiently import long-chain fatty acids would be predicted to be less affected. We thus tested an isogenic *Salmonella fadL* mutant; this disruption prevented transport of these compounds,^11^ presumably c2-HDA among them. For untreated *Salmonella*, we found the proportion of the *sicA*-expressing population to be 80%, while co-cultured with recombinant *E. coli* expressing *rpfF* reduced that to only 53%, comparable to the effect of the non-recombinant *E. coli* control strain. These findings thus demonstrate *that E. coli* Nissle itself can modestly affect *Salmonella* invasion, but that c2-HDA, when recombinantly produced, reduces the proportion of the *Salmonella* population capable of expressing invasion genes and does so in a way consistent with its action as a cytoplasmic long-chain fatty acid signal.

### Recombinant *E.* *coli* can stably colonize the chicken intestine and produce c2-HDA

**Figure 5.**
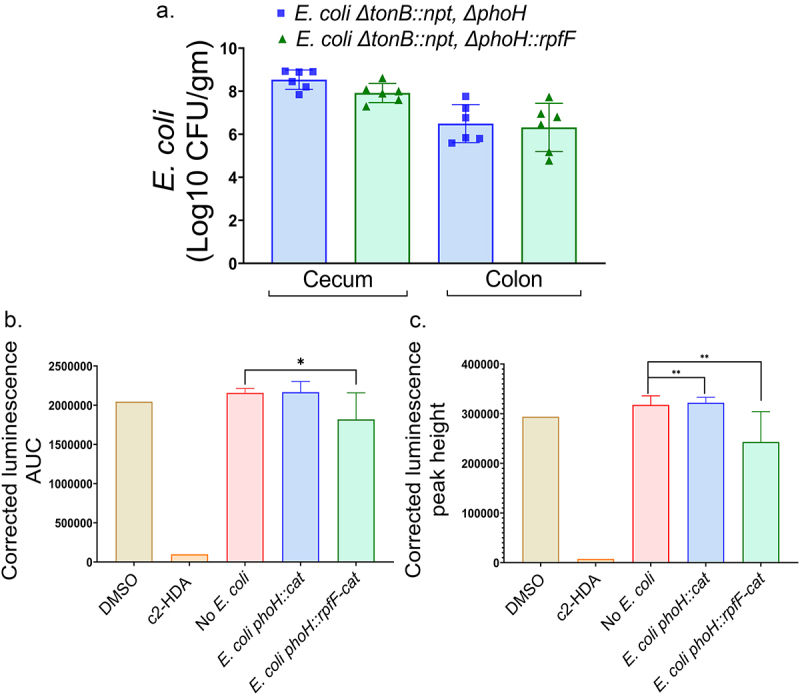
Recombinant *E.* *coli* can stably colonize the chicken intestine and produce c2-HDA. (a) Chicks were orally inoculated on day two of age with *E.* *coli* Nissle 1917 Δ*tonB* strains expressing or lacking *rpfF*, followed on day four of age by *Salmonella* inoculation, with *E.* *coli* numbers in the cecum and colon determined on day 13 of age. (b) Fatty-acid extracts of colon contents were added to cultures of *S.* Typhimurium carrying a plasmid-borne *hilA-luxCDABE* reporter fusion, and *hilA* expression was measured by luminescence as a function of culture turbidity. Shown here are the mean peak expression and area under the curve (1 to 24 hours) for each condition. DMSO and c2-HDA (2 µM) were used as controls. Error bars show standard deviation (*n* = 3).

To determine whether these recombinant strains could colonize an agricultural animal important to the spread of *Salmonella*, we used a chicken model, comparing the carriage of *E. coli* Nissle 1917 to that of an isogenic mutant expressing *rpfF*. As a stringent test of colonization, we used a *tonB* mutant in this case, as, in a murine model, wild type *E. coli* Nissle 1917 has been reported to itself reduce *Salmonella* colonization by competition for iron, an advantage negated by the loss of *tonB*.^26^ Chicks were inoculated at 2 days of age with approximately 1 × 10^8^ cfu of the *E. coli* Nissle 1917 strains and then followed on day 4 of age with a large inoculum of approximately 1 × 10^8^ cfu *Salmonella* serovar Enteritidis. On day 13 of age, chicks inoculated with either strain carried large numbers of *E. coli* in their large intestines (Figure 5a). Both groups maintained more than 10^8^ cfu/gram of intestinal contents in the cecum and 10^7^ cfu/gram in the colon. To next determine whether an *rpfF*-expressing strain could produce a compound repressive to *Salmonella* invasion while residing in the gut, we performed fatty acid extractions on the gut contents of chickens inoculated with the recombinant *E. coli* and its isogenic non-recombinant strain, along with an uninfected control group given only PBS. These extracts were added to our reporter strain of *Salmonella* growing in laboratory medium, again assessing the expression of the *hilA-luxCDABE* fusion (Figure 5b). We found that samples taken from five of six chickens that had received the recombinant *rpfF*-expressing *E. coli* strain reduced *hilA* expression, as measured by both peak expression and area under the curve and that in aggregate this group significantly reduced expression of this *Salmonella* invasion gene. In contrast, comparable samples derived from chickens receiving either the non-recombinant strain without *rpfF* or no *E. coli*, produced no effect. These results therefore demonstrate that *rpfF* can be recombinantly expressed in a well-characterized *E. coli* strain that stably colonizes the gut of chickens and there produces a fatty-acid signal capable of repressing *Salmonella* invasion.

### Recombinant *E.* *coli* reduces *Salmonella* colonization of the chicken cecum

**Figure 6.**
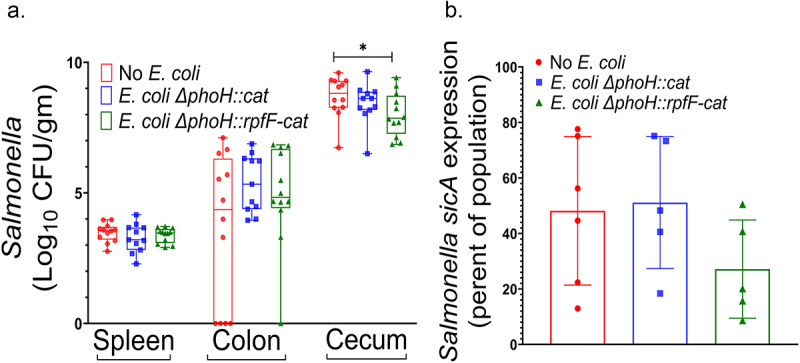
Recombinant production of c2-HDA by *E.* *coli* reduces *Salmonella* colonization of the chicken cecum. A. Chickens were administered recombinant *E.* *coli* expressing *rpfF*, the isogenic *E.* *coli* strain without *rpfF*, or a PBS control on days 1, 3, and 6 of life and were given a *S.* Enteritidis reporter strain (Δ*phoN*:BFP, *sicA*-GFP) on day 4 of life. On day 13, the spleen and contents of the cecum and colon were analyzed by selectively plating to determine the number of *Salmonella* present. The experiment was performed twice, each time with 6 chickens/group, for total *n* = 12. Median is shown by the central bar; boxes signify 25% and 75% quartiles, and vertical bars show the range. B. Contents of the cecum and colon were analyzed by flow cytometry to determine the proportion of the Salmonella population expressing sicA. Error bars show standard deviation (*n* = 5–6).

*Salmonella* is highly adapted to colonize the intestine of poultry, a function dependent upon multiple bacterial virulence determinants. As a proof of concept, we asked whether the direct delivery of c2-HDA, through its recombinant production in the gut by *rpfF*-expressing *E. coli*, could alter that colonization. For these experiments, we used wild type *E. coli* Nissle 1917 as the background strain, in an attempt to achieve maximal colonization of the gut. On days 1, 3, and 6 of life, we orally administered to chickens ~1 × 10^8^ of either *E. coli* Nissle 1917 expressing *rpfF*, an isogenic non-recombinant *E. coli* Nissle 1917 strain, or a PBS control. On day 4, these chickens were given ~1 × 10^6^ *S*. *enteritidis*, again using a reporter strain constitutively producing BFP and expressing GFP under the control of the chromosomal *sicA* promoter, and colonization of abdominal organs was assessed on day 13 of life (Figure 6a). Low numbers of *Salmonella* were detected in the spleen, with means of 2.8 × 10^3^ and 4.0 × 10^3^/gram of tissue, and these were not different among the three groups. Similarly, colons yielded 1.5 × 10^6^ to 1.8 × 10^6^/gram of contents, also not differing among the groups. Within the ceca, however, we found high numbers of *Salmonella*, ranging from 4.4 × 10^8^ to 1.1 × 10^9^ among the three groups. *Salmonella* colonization was reduced significantly in chickens receiving the *rpfF*-expressing *E. coli*, with a mean reduction of 2.6-fold and a median reduction of 10-fold when compared to PBS-control group, while colonization of chickens receiving the non-recombinant *E. coli* did not differ significantly from that of the PBS control. To determine whether the biological production of c2-HDA could affect *Salmonella* invasion within the complex milieu of the intestine, we used this same experimental protocol, analyzing contents of the cecum and colon by flow cytometry to determine the proportion of the *Salmonella* population expressing *sicA* (Figure 6b). We detected a mean expression of this gene in 48% of the *Salmonella* population from chickens receiving PBS and in 51% of those treated with the non-recombinant *E. coli* strain, but 27% of the *Salmonella* population from chickens that had received the *rpfF*-expressing *E. coli* strain expressed this invasion gene. Due to variability among the individual animals, however, this difference did not reach the level of statistical significance. These results thus demonstrate that c2-HDA production can reduce the survival and proliferation of *Salmonella* within an animal host.

## Discussion

Here we have tested a novel means to reduce the virulence of *Salmonella* by impairing invasion, the penetration of the intestinal epithelium by this pathogen, using the potent chemical inhibitor c2-HDA. The concept of controlling bacterial pathogens by affecting their virulence is not new, but invasion, as a function required for both colonization and disease in animals, provides a particularly attractive target for intervention. It has indeed long been known that fatty acids of various classes can reduce invasion and, in some cases, have demonstrated their efficacy.^11–13,31–34^ However, the practical use of these chemical signals remains problematic. As fatty acids are rapidly absorbed through the brush border of the small intestine, only a small fraction of an administered dose may reach the large intestine,^35^ the site of *Salmonella* colonization and growth, thus making treatment through oral administration inefficient and costly. We have attempted to circumvent this problem by the production of a fatty-acid signal *in situ*, within the large intestine, through its production by a recombinant *E. coli* capable of sustained colonization of that organ and have demonstrated the efficacy of this approach using a chicken model. Equally important to these efforts is the use of a chemical signal of high potency. A wide range of fatty acids can be used to reduce *Salmonella* virulence, but most require concentrations in the millimolar range to do so.^12,34^ In contrast, c2-HDA is the most potent thus far described, reducing the expression of invasion genes when supplied at sub-micromolar concentrations,^20^ and therefore potentially improving its effects within the complex chemical and biological environment of the gut.

As with any proposed means to control bacterial pathogens, the efficient selection of resistant mutants presents an imposing hurdle. We have ourselves, in fact, identified point mutants of HilD, one of the targets of c2-HDA, that make this central invasion regulator fully refractory to the effects of chemical binding and inhibition,^21^ suggesting that such resistance may rapidly develop. Several lines of evidence, however, both theoretical and practical, argue against this outcome. c2-HDA is present in the gut,^21^ presumably sensed by *Salmonella* as an *in vivo* signal to modulate its virulence and optimize its survival. As the uncontrolled expression of invasion is detrimental to *Salmonella* growth, mutants that exhibit resistance to c2-HDA will likely themselves suffer fitness defects within the competitive environment of the gut, and thus not become fixed in the population. Equally important is the fact that invasion is an altruistic function. The specific *Salmonella* bacteria that invade die in the process,^36^ but in doing so aid in the survival of their population by creating a gut environment that is conducive to *Salmonella* growth.^37–39^ Thus, mutants insensitive to c2-HDA, and therefore hyper-invasive, are also destined to be lost from the population. Finally, c2-HDA inhibits not just one regulator of invasion, but at least three: HilD, HilC, and RtsA.^20,22^ Although the *in vitro* effects of *hilD* mutants alone are clear, the possibility of redundant *in vivo* functions reduces the possibility of evolving fully insensitive mutants.

The ability of c2-HDA to reduce *Salmonella* colonization of the chicken demonstrated here is admittedly quite modest: reducing carriage to the extent we observed is unlikely to have discernable commercial consequences. We will note, however, that the experimental model employed was necessarily rigorous and perhaps overly so. To obtain consistent infection with manageable group sizes, we used a *Salmonella* inoculum that produced a cecal colonization of ~10^9^/gram of contents. Natural infections in chickens, however, are much less severe, averaging less than 10^3^/gram.^40^ It is thus plausible that an intervention strategy yielding a similar reduction in naturally infected poultry will have more meaningful effects, a supposition that will require further investigation.

## Materials and methods

### Strains, plasmids, and growth conditions

*Salmonella enterica* subsp. *enterica* serovar Typhimurium 14028s and *Salmonella enterica* subsp. *enterica* serovar Enteritidis MD15, and mutants thereof were used throughout, as listed in Supplementary Table S1. *E. coli* DH5α was used for cloning and plasmid maintenance, while *E. coli* Nissle 1917 was used for gene integration and animal studies. *rpfF* orthologs were codon-optimized using OPTIMIZER (http://genomes.urv.es/OPTIMIZER/) with the One Amino Acid One Codon approach and synthesized in pUC57 (BioBasic) under the control of the *tacI* promoter.^41^ Sources of *rpfF* sequences are shown in Supplementary Table S1. Mutant strains were constructed using the one-step inactivation method as previously described.^42^ *rpfF* integrated into the *E. coli* chromosome was cloned under the control of the P3 promoter or the P2 promoter, reported to provide high and medium expressions, respectively,^23^ obtained from Addgene. All bacterial cultures were grown in glass test tubes (18 mm × 150 mm) at 37°C with aeration (210 rpm) unless otherwise described.

### Luciferase assays

*Salmonella* Typhimurium carrying a *hilA-luxCDABE* reporter fusion plasmid and *E. coli* strains were grown overnight in LB (5 g/L NaCl, yeast extract 5 g/L and tryptone 10 g/L) with appropriate antibiotics, at 37°C with aeration. These were then diluted at 1:100 and grown for ~16 hours in M9 Minimal medium (8.5 mM NaCl, 47.7 mM Na_2_HPO4. 7 H_2_O, 22 mM KH_2_PO_4_, 18.7 mM NH_4_Cl, 2 mM MgSO_4_, and 100 µM CaCl_2_) with 0.2% glucose and appropriate antibiotics. *Salmonella* was inoculated at an OD_600_ of 0.02 with extracted fatty acids or equal volume of DMSO. For co-culture assays, *Salmonella* was inoculated at an OD_600_ of 0.02 along with an equal volume of *E. coli* at an OD_600_ of 0.002 in LB with appropriate antibiotics to achieve a ratio of 10:1 into black-walled 96 well plates. Luminescence and turbidity were measured every 30 minutes for 24 hours at 37°C aerobically using a Biotek Synergy H1 microplate reader, and measurements were corrected for culture density by dividing luminescence by turbidity. All strains and conditions were tested in replicates of at least three.

### Fatty acid extraction

Overnight cultures in LB at 37°C with aeration were centrifuged, and the supernatant was collected. An equal volume of chloroform was added to the supernatant and vortexed at maximum speed for 20 seconds, chloroform was removed, and the extraction process was repeated five times. The organic phases were combined, and chloroform was allowed to evaporate at room temperature. After complete evaporation, the organic contents were dissolved in a small volume of DMSO to allow the purification of fatty acids by Dole extraction.^43^ Briefly, the evaporated organic contents were resuspended in 500 µl of DMSO and vortexed. Five volumes of Dole extraction mix (Isopropanol: heptane: sulfuric acid 39:10:1) were added and vortexed, followed by the addition of two volumes of water and six volumes of heptane. The mixture was thoroughly vortexed and allowed to rest for separation of aqueous and organic phases. A separate organic phase was collected and evaporated at room temperature. Dried extracts of fatty acids were dissolved in 100 µl DMSO. To extract fatty acids from chicken intestine, colon contents were diluted in PBS, vortexed thoroughly, and passed through a 5 µm filter to remove large debris. Organic contents from the filtrates were purified as described above and were dissolved in 100 µl DMSO.

### Liquid chromatography–mass spectrometry

Bacterial cultures in duplicate were grown in LB at 37°C aerobically for 10 hours and centrifuged. Supernatants were collected, and organic contents were separated by chloroform extraction as described above. Chloroform evaporated at room temperature, and dried extracts were dissolved in acetone and subjected to LC-MS analysis. As an internal control, c2-HDA was added to samples with known concentrations. Linoleic acid and dihomo-gamma-linoleic acid were used as standards and prepared individually. Fatty acids were detected in negative ion mode and structures were confirmed by MS/MS spectrum.

### β-galactosidase assays

*Salmonella* Typhimurium Δ*hilD*, Δ*rtsA*, Δ*hilC* strains with a *sipB:lacZY* fusion and plasmids expressing differing *hilD* alleles (wild type, HilD^N44A^, HilD^K293A^, or HilD^Q290A^ mutant)^21^ under the control of a tetracycline-inducible promoter were grown in 1 ml of LB with 100 mM MOPS and induced to wild-type levels with 1 µg/ml tetracycline, to which was added 10 µl of fatty-acid extract obtained from *E. coli* strains or DMSO as the control. Cultures were then grown for 16 hours without aeration at 37°C, and β-galactosidase assays were performed as described.^44^ All strains and conditions were tested in replicates of four.

### Invasion assay

Experiments were performed as described^45^, with variations. *Salmonella* and *E. coli* strains were grown overnight in LB with aeration at 37°C, and densities were measured by spectrophotometry. These were then co-cultured in LB with an initial ratio of 1:1 or 10:1 *Salmonella* to *E. coli* and grown to the OD_600_ of ~1.0 at 37°C with aeration As controls, *Salmonella* was grown in LB without *E. coli*, in the presence of 20 µM c2-HDA or equal volume of DMSO. HEp-2 monolayers in 24-well cell culture plates (~5 × 10^5^ were infected with ~5 × 10^6^ cfu bacteria [1:10 MOI]). Contact of bacteria with the monolayer was facilitated by centrifugation at 500 × *g* for 10 minutes. Infected cells were incubated at 37°C with 5% CO^2^ for 1 hour to allow internalization. The non-invading bacteria were removed by washing three times with PBS, and the cells were incubated with media containing 200 µg/ml gentamicin for an hour. Following incubation, the cells were lysed by 1% Triton-X 100, and dilutions were spread on agar plates with appropriate antibiotics. Additionally, to enumerate the exact number of *Salmonella* and *E. coli* in the inoculum, dilutions of the inoculum were spread on agar plates. Invasion rates were calculated by dividing the number of recovered *Salmonella* to that present in the inoculum.

### Flow cytometry

*Salmonella* Typhimurium *fadL*^*+*^ and Δ*fadL* strains harboring constitutively expressed BFP and *sicA*-GFP (Δ*phoN*::BFP, *sicA*-GFP)^2^ were co-cultured in LB at 37°C with aeration and 1 mM nonanoic acid, with isogenic *E. coli* Nissle 1917 Δ*tonB* strains expressing or lacking *rpfF*. All pairs of strains were diluted to an OD_600_ of 0.1, inoculated in equal volumes, and grown together for 6 hours in LB with an aeration at 37°C. Samples were prepared for flow cytometry by centrifugation and resuspension in 1 ml of 4% paraformaldehyde in 1× PBS and fixed for 30 minutes in ice. The cells were then pelleted to remove paraformaldehyde and resuspended in 1× PBS. Recovered cells were analyzed for blue fluorescent protein (BFP) and green fluorescent protein (GFP) expression using an Attune analyzer NxT flow cytometer (Thermo Fisher). *Salmonella* was identified by BFP expression, and GFP was used to monitor *sicA* expression, with the proportion of the *sicA*-expressing population calculated as BFP+/GFP+ particles divided by the total BFP+ population.

### Animal experiments

To assess *E. coli* colonization of the chicken gut, mixed-sex white leghorn chicks were randomly assigned on the day of hatch into groups of 5–6, maintained in separated brooders, and provided with antibiotic-free feed *ad libitum*. On day 2 of age, two groups were given ~1 × 10^8^ cfu in 100 µl of PBS of *E. coli* by oral gavage: one received *E. coli* Nissle 1917 Δ*tonB*, and the other an isogenic strain expressing *C. turicensis rpfF* under the control of a medium-strength promoter,^23^ integrated into the chromosome at *phoH*. Both strains were marked by kanamycin resistance at *tonB*. These bacteria were grown in LB at 37°C with aeration for 16 hours. A third group was gavaged with PBS as a control to ensure that *E. coli* produced no untoward health effects. On day 4 of age, all chicks were given ~1 × 10^8^ cfu *Salmonella* serovar Enteritidis by oral gavage grown for 16 hours in LB at 37°C with aeration. On day 13 of age, chicks were euthanized, and contents of ceca (one per chick) and colons were harvested. Serial dilutions of samples were made in PBS and were plated onto LB agar with kanamycin (50 µg/ml) to enumerate *E. coli* populations. To identify invasion-repressing signals produced within the chicken intestine, organic extractions were performed using equal amounts of colon contents from chickens inoculated with the recombinant *rpfF*-expressing *E. coli* or its isogenic non-recombinant strain, or with the PBS control, using the extraction method described. These extracts were added to a reporter strain of *Salmonella* (10 µl of 1 ml of culture, or a comparable DMSO control), measuring the expression of the *hilA-luxCDABE* fusion as described above. To determine whether expression of *Salmonella* invasion was inhibited in the chicken gut, groups of six chicks were given by oral gavage either ~1 × 10^8^ *E. coli* Nissle 1917 expressing *rpfF*, or an isogenic non-recombinant strain (otherwise wild type), or PBS on days 1, 3, and 6 of life. All the chicks received ~1 × 10^6^ *S*. Enteritidis *ΔphoN*::BFP, *sicA*-GFP on day 4 of life by oral gavage. At 13 days of life, chicks were euthanized and their cecal contents were analyzed for *sicA*::GFP expression by flow cytometry as described above. To determine colonization rates, this same experimental protocol was used. Spleens, colon contents and cecal contents were serially diluted in PBS and plated onto LB agar with either chloramphenicol, for selection of *E. coli*, or kanamycin, for selection of *Salmonella*. This experiment was repeated, and the data were combined, providing 12 chickens/treatment group.

### Statistical analysis

For all the assays, differences among groups were determined using the Mann–Whitney test, with *p* values <0.5 being considered significant.

### Ethics statement

Studies involving vertebrate animals were approved by Cornell University's Institutional Animal Care and Use Committee (Protocol 2012–0074). Euthanasia was performed using carbon dioxide inhalation in accordance with the American Veterinary Medical Association Guidelines for Euthanasia of Animals. The Cornell University Animal Care and Use program and associated animal facilities are operated in accordance with the U.S. Department of Agriculture Animal Welfare Act (1966), Regulation (C.F.R., 2009) and policies, the Health Research Extension Act (1985), the Public Health Service Policy on Humane Care and Use of Laboratory Animals (PHS, 2002), the Guide for the Care and Use of Laboratory Animals (NRC, 2011), the Guide for the Care and Use of Agricultural Animals in Research and Teaching (2010), the New York State Health Law (Article 5, Section 504), and other applicable federal, state, and local laws, regulations, policies, and guidelines.

### Health and safety

All work described here was conducted in accordance with laboratory health and safety procedures approved by the Cornell University Institutional Biosafety Committee. This includes protocols for growing, handling, and transporting pathogenic bacterial organisms, as well as safety training for all laboratory personnel.

## Supplementary Material

Supplemental Material

## Data Availability

The data that support the findings of this study are openly available in Cornell University Library’s institutional repository, eCommons (https://ecommons.cornell.edu), for preservation and access (https://doi.org/10.7298/gv1c-b060). Datasets will be available via the world-wide web without restriction. eCommons provides each item with a persistent identifier and is committed to preserving the binary form of the digital object.
