# Supplementary material for: Recombinant production of a diffusible signal factor inhibits Salmonella invasion and animal carriage
Source: Gut Microbes. 2023 May 9;15(1):2208498. doi: 10.1080/19490976.2023.2208498 (PMC10171134; doi:10.1080/19490976.2023.2208498)
Supplement: Supplemental Material [file KGMI_A_2208498_SM3468.docx]

**Table S1. Strains and plasmids used in this study**

| **Strain** | **Description** | **Reference** |
| --- | --- | --- |
| 14028s | *S.* Typhimurium wild type | Laboratory Collection |
| CA2286 | *S.* Typhimurium/p*hilA-luxCDABE* | ^12^ |
| CA2946 | *S.* Typhimurium/p*hilA-luxCDABE* | ^12^ |
| CA4796 | *S.* Typhimurium *sipB::lacZY*, *recD::Tn10,* ,Δ*hilD::cat,* Δ*hilC,* Δ*rtsA,* pHilD | ^21^ |
| CA4881 | *S.* Typhimurium *sipB::lacZY*, *recD::Tn10,* ,Δ*hilD::cat,* Δ*hilC,* Δ*rtsA* pHilD^N44A^ | ^21^ |
| CA4857 | *S.* Typhimurium *sipB::lacZY*, *recD::Tn10,* ,Δ*hilD::cat,* Δ*hilC,* Δ*rtsA* pHilD^K293A^ | ^21^ |
| CA4856 | *S.* Typhimurium *sipB::lacZY*, *recD::Tn10,* ,Δ*hilD::cat,* Δ*hilC,* Δ*rtsA* pHilD^Q290A^ | ^21^ |
| CA4886 | *S.* Typhimurium *sipB::lacZY*,Δ*hilD*,Δ*hilC*,Δ*rtsA* pWSK29 | ^21^ |
| MD15 | *S.* Enteritidis wild type | ^2^ |
| CA3887 | *S.* Enteritidis MD15 Δ*phoN*::BFP,*sicA*-GFP | ^2^ |
| CA5475 | *S.* Enteritidis MD15 Δ*phoN*::BFP,*sicA*-GFP,Δ*fadL* | This study |
| CA5484 | *S.* Enteritidis MD15 Δ*phoN*::BFP,*sicA*-GFP,Δ*malXY::npt* | This study |
| DH5α | *E. coli* K12 | Laboratory Collection |
| Nissle 1917 | *E. coli* wild type | Pharma-Zentrale |
| CA5206 | *E. coli* Nissle 1917 Δ*phoH::rpfF-cat* (P2 promoter) | This study |
| CA5218 | *E. coli* Nissle 1917 Δ*phoH::rpfF-cat* (P3 promoter) | This study |
| CA5197 | *E. coli* Nissle 1917 Δ*phoH::cat* | This study |
| CA5227 | *E. coli* Nissle 1917 Δ*tonB::npt*, Δ*phoH::rpfF* (P2 promoter) | This study |
| CA5226 | *E. coli* Nissle 1917 Δ*tonB::npt*, Δ*phoH* | This study |
|  |  |  |
| **Plasmid** | **Description** | **Reference** |
| pBA426 | p*hilA-luxCDABE* | ^12^ |
| pCA234 | *cat* from pKD3 in pUC57 | This study |
| pCA248 | *rpfF* ortholog of *C. turicensis* (TWR33075) in pCA234 | This study |
| pCA247 | *rpfF* ortholog of *X. fastidiosa* (AAO28287) in pCA234 | This study |
| pCA249 | *rpfF* ortholog of *X. campestris* (AAM41146) in pCA234 | This study |
| pCA250 | *rpfF* ortholog of *S. maltophilia* (ABD59453) in pCA234 | This study |
| pCA251 | *rpfF* ortholog of *P. aeruginosa* (NP_249436) in pCA234 | This study |
| pFAB3677 | P2 promoter | ^23^ |
| pFAB3689 | P3 promoter | ^23^ |
| pHilD | pWSK29-*tetRA*-*hilD*-3XFLAG | ^21^ |
| pHilD^N44A^ | pWSK29-*tetRA*-*hilD*^N44A^-3XFLAG | ^21^ |
| pHilD^K293A^ | pWSK29-*tetRA*-*hilD*^K293A^-3XFLAG | ^21^ |
| pHilD^Q290A^ | pWSK29-*tetRA*-*hilD*^Q290A^-3XFLAG | ^21^ |
| pWSK29 | pWSK29 | ^21^ |
